# Depression and the risk factors for isolated infectious disease fever patients in the hospital during the COVID-19

**DOI:** 10.12669/pjms.39.2.6902

**Published:** 2023

**Authors:** Guohui Jin, Lei Li, Jing Liu, Baoyan Wang, Bo Zhan

**Affiliations:** 1 Guohui Jin, Department of Infectious Disease, Baoding No.1 Hospital, Baoding 071000, Hebei, China; 2 Lei Li, Department of Infectious Disease, Baoding No.1 Hospital, Baoding 071000, Hebei, China; 3 Jing Liu, Department of Rheumatology, Baoding No.1 Central Hospital, Baoding 071000, Hebei, China; 4 Baoyan Wang, Department of Infectious Disease, Baoding No.1 Hospital, Baoding 071000, Hebei, China; 5 Bo Zhan, Department of Infectious Disease, Baoding No.1 Hospital, Baoding 071000, Hebei, China

**Keywords:** Infectious Disease, Hospitalized Isolation, Home Isolation, Anxiety, Depression

## Abstract

**Objectives:**

To compare and analyze the incidence of anxiety and depression of infectious disease fever patients in hospitalized isolation and home isolation during the COVID-19 pandemic, as well as the risk factors for the negative emotions of hospitalized isolation patients.

**Methods:**

Forty isolated infectious disease fever patients in Baoding No.1 Hospital were randomly selected as the study group, and the other 40 isolated infectious disease fever patients at home were randomly selected as the control group from March 2020 to August 2020. The scores and prevalence of depression and anxiety between the two groups were compared and analyzed. The logistic regression analysis was used to judge and analyze the negative psychological factors of hospitalized isolation patients such as depression and anxiety.

**Result:**

The HAMA and HAMD-17 scores of study group are significantly higher than those of control group (HAMA, p=0.00; HAMD-17, p=0.01). The prevalence of anxiety and depression in the study group was significantly higher than that in the control group (p=0.03, p=0.04). The gender (p=0.002), economic status (p=0.004) and isolation attitude (p=0.023) are the related factors of anxiety, among which economic status is the protective factor, while women and resistant attitude are the risk factors. Economic status (p=0.003) and isolation attitude (p=0.001) are the related factors of depression, among which economic status is the protective factor, and resistant attitude is the risk factor.

**Conclusion:**

The prevalence and severity of anxiety and depression in hospitalized isolation patients due to infectious disease fever are significantly higher than those of home isolation patients. The focus groups are women, with bad economic status and poor isolation attitude. Necessary psychological counseling and social support should be provided to these groups to reduce negative emotions and increase the experience of isolated patients.

## INTRODUCTION

COVID-19 is an epidemic infectious disease endangering public health at present, with the characteristics of rapid transmission, multiple transmission routes, strong infectivity and general susceptibility among the public.^1^ During the spread of the epidemic and the normalization of prevention and control, infectious disease patients who have fever symptoms need to be isolated for observation until COVID-19 is eliminated by screening. Isolation can cause dramatic changes in patients’ psychology^2^, and anxiety and depression symptoms are common in this special group.^3^ At present, the isolation measures adopted are divided into hospitalized isolation and home isolation. In this study, patients with infectious diseases were divided into the hospitalized isolation group and the home isolation group.

Hamilton Anxiety Scale (HAMA), Hamilton Depression Scale (HAMD-17) and the general data were used to investigate and evaluate the anxiety and depression levels of the two groups so as to clarify their anxiety and depression conditions. By comparing and analyzing the differences in the occurrence of anxiety and depression between the two groups, the multivariate logistic regression analysis was carried out on the hospitalized isolation population to clarify the factors of anxiety and depression in the group. While helping clinical medical staff in disease prevention and treatment, it also aims to provide guidance for improving patients’ anxiety and depression symptoms and shortening the course of the disease.

## METHODS

Forty isolated infectious disease fever patients in Baoding No.1 Hospital were randomly selected as the study group, and the other 40 isolated infectious disease fever patients at home were randomly selected as the control group from March 2020 to August 2020. In the study group, there were 23 males and 17 females aged from 25 to 64 years old, with an average of 44.54 ± 17.32 years old; in the control group, there were 21 males and 19 females aged from 28 to 70 years old, with an average of 45.12 ± 16.41 years old. There was no significant difference in the comparison of the general data between the two groups, and the two groups were comparable (Table-I). The study was approved by the Institutional Ethics Committee of Baoding No.1 Hospital (No.:2022022207; Date: February 22, 2022), and written informed consent was obtained from all participants.

**Table-I:** Comparison and analysis of the general data between the study group and the control group (*χ̅*± *s*) n=40.

Indicators	Study group	Control group	t/χ^2^	P
Age	44.54±17.32	45.12±16.41	0.15	0.88
Male	23	21	0.20	0.65
Married	32	28	0.17	0.30
Educational Level				
Below university level	15	13	0.22	0.64
University level	16	18	0.20	0.65
Above university level	8	9	0.07	0.79
Economic status				
Good	7	8	0.08	0.76
Common	23	21	0.20	0.65
Poor	10	11	0.06	0.80
Isolation attitude			0.25	0.62
Favorable	28	30		
Opposed	12	10		

P>0.05.

### Inclusion criteria:

- Meet the diagnostic criteria of infectious diseases^4^, the body temperature is ≥37.3 °C, and it is necessary to be isolated for observation;
- Age <75 years old;
- Informed consent and sign the informed consent form.

### Exclusion criteria:

- Patients with severe liver, kidney and other organ dysfunctions, and they cannot be satisfactorily controlled;
- Patients with consciousness disorders;
- Patients with mental disorders and cognitive impairment, and they cannot cooperate to complete the study.

The HAMA scale, developed by Hamilton, was clinically used to assess anxiety. With the 5-level scoring method, its 14 items could well reflect the severity of anxiety, and each score is added to form a total score. A total score of 8 to <14 indicated anxiety tendency, and a total score of ≥14 indicated anxiety. 5

The HAMD-17 scale, developed by Hamilton, was clinically used to assess depression. There were 17 items on the scale, and the 5-level scoring method was used. Each score was added to form a total score. A total score of eight to <fourteen indicated a tendency to depression, and a total score of ≥ 14 indicated depression.^6^

### Statistical analysis:

All data were analyzed by SPSS 20.0 software, and the measurement data are expressed in (± s). The data between groups are analyzed by the t-test of two independent samples, and the related risk factors are analyzed by the logistic regression analysis. P< 0.05 means that the difference is statistically significant.

## RESULTS

The HAMA and HAMD-17 scores of hospitalized isolation patients are significantly higher than those of home isolation patients, and the difference is statistically significant (HAMA, p=0.00; HAMD-17, p=0.01) (Table-II).

**Table-II:** Comparative analysis of inflammatory factor changes before and after treatment in the two groups (*χ̅*± *s*) n=40.

Category	HAMA Score*	HAMD-17 Score*
Study group	15.73±2.76	17.24±4.82
Control group	13.06±3.08	14.16±5.07
t	4.10	2.87
p	0.00	0.01

* p<0.05.

The prevalence of anxiety in hospitalized isolation patients was 77.5%, and that in home isolation patients was 57.5%. The study group is significantly higher than the control group, and the difference is statistically significant (p=0.03); the prevalence of depression was 62.5% in the study group and 40% in the control group, with a statistically significant difference (p=0.04) (Table-III and Table-IV).

**Table-III:** Comparative analysis of the anxiety incidence rate between the two groups of isolation patients (*χ̅*± *s*) n=40.

Category	Mild (cases)	Moderate (cases)	Severe (cases)	Incidence*
Study group	16	11	4	31(77.5%)
Control group	12	9	2	23(57.5%)
χ^2^				4.71
p				0.03

* p<0.05.

**Table-IV:** Comparative analysis of the depression incidence rate between the two groups of isolation patients (*χ̅*± *s*) n=40.

Category	Mild (cases)	Moderate (cases)	Severe (cases)	Incidence*
Research group	14	9	2	25(62.5%)
Control group	9	6	1	16(40%)
χ^2^				4.05
p				0.04

* p<0.05.

Taking the general data such as age, gender, marriage, education level, economic status and isolation attitude as the independent variables, a multivariate logistic regression analysis was carried out after assignment, α in=0.05, α Out=0.10. The results show that gender (p=0.002), economic status (p=0.004) and isolation attitude (p=0.023) are the related factors of anxiety, among which economic status is the protective factor, while women and resistant attitude are the risk factors (Table-V).

**Table-V:** The multivariate logistic regression analysis on anxiety-related factors of hospitalized isolation patients n=40

Dependent variables	β-value	SE value	Waldχ^2^	P-value	O value	95.0% CI
Age (control group = < 30 years old)	0.923	0.657	1.473	0.075	2.543	0.237~4.620
Gender (control group = male) *	-1.187	0.642	4.125	0.002	2.307	-1.254~3.801
Marriage (control group = unmarried)	0.197	0.285	0.972	0.473	0.826	0.571~1.638
Economic status (control group = poor) *	1.157	0.563	4.193	0.004	3.175	1.037~8.022
Education level (control group = below university)	0.607	0.384	2.447	0.283	1.832	0.782~3.275
Isolation attitude (control group = favorable) *	0.012	0.626	2.907	0.023	0.275	0.081~0.873

* p<0.05.

With the general data such as age, gender, marriage, education level, economic status and isolation attitude as the independent variables, a multivariable logistic regression analysis was carried out after assignment, α In =0.05, α Out =0.10. The results show that economic status (p=0.003) and isolation attitude (p=0.001) are the related factors of depression, among which economic status is the protective factor and resistant attitude is the risk factor (Table-VI).

**Table-VI:** The multivariate logistic regression analysis on depression-related factors in hospitalized isolation patients n=40

Dependent variables	Β value	SE value	Waldχ^2^	P value	O value	95.0%CI
Age (control group = < 30 years old)	0.080	0.126	1.072	0.306	1.090	0.932~1.287
Gender (control group = male) *	-0.047	0.095	0.267	0.612	0.960	0.832~1.133
Marriage (control group = unmarried)	0.188	0.127	2.141	0.158	1.193	0.964~1.550
Economic level (control group = poor) *	0.497	0.163	9.703	0.003	1.642	1.220~2.236
Education level (control group = below university)	0.937	0.157	12.047	0.001	0.397	0.302~1.272
Isolation attitude (control group = approval) *	0.447	0.184	12.001	0.154	1.192	0.982~1.230

* p<0.05.

## DISCUSSION

Isolated medical observation refers to the measures taken to isolate and limit the scope of activities for those who may be exposed to infectious sources. By carrying out symptom monitoring, it aims to make an early diagnosis, reduce the risk of infecting others and avoid the spread of infectious diseases among the public.^7^ In recent years, it has played an active role in public health emergencies such as SARS and MERS. However, previous studies have found that due to the factors such as separation from relatives, helplessness and the fear of being infected with infectious diseases during isolation, those isolated for observation have psychological symptoms such as anxiety, depression, insomnia and post-traumatic stress, and some psychological problems are characterized by persistence and complexity.^8^ Therefore, while taking isolation measures, we should try to reduce or avoid the negative impact on the mental health of those isolated for observation.^9^

The current isolation measures mainly include home isolation and hospitalized isolation. This study shows that the HAMA and HAMD-17 scores of hospitalized isolation patients are significantly higher than those of home isolation patients (HAMA, p=0.00; HAMD-17, p=0.01); The prevalence rate of anxiety in hospitalized isolation patients was 77.5%, and that in home isolation patients was 57.5%. The study group is significantly higher than the control group, and the difference is statistically significant (p=0.03); The prevalence of depression was 62.5% in the study group and 40% in the control group, with a statistically significant difference (p=0.04).

It is suggested that the anxiety and depression of those isolated for observation in the hospital are high, proving that home isolation has visible advantages over hospitalized isolation. It is more conducive to the mental health of those isolated for medical observation. The research results of Carriedo et al.^10^ show that people who often conduct VPA (vigorous physical activity) and MVPA (moderate-to-vigorous physical activity) during isolation have higher positive emotion scores and lower depression symptom scores. In this regard, home isolation has significant advantages. Ranasinghe et al.^11^ also believe that it is reasonable to improve host immunity and reduce the adverse effects of isolation through physical activity, and MVPA should be encouraged during and after the current COVID-19 pandemic.

But Şenışık deems that athletes with better mental health than ordinary people have the opposite negative effect after resting all the time during isolation.^12^ Positive emotions are related to the development of creative and comprehensive activities. In the case of negative emotions, the family environment has a dominant protective position. A good family environment can promote mental health during the COVID-19 pandemic.^13^ Therefore, the prevalence of anxiety and depression in home isolation patients is significantly lower than that in hospitalized isolation patients.

There is a correlation between basic emotions and Maslow’s Hierarchy of Needs. Emotional patterns are made up of positive emotions, negative emotions and proud emotions. Both positive emotions and proud emotions can resist negative emotions.^14^ In times of crisis, fear, anger and sadness dominate all groups. Anger and disgust mainly occur when people are faced with the risk of failing to meet their survival needs, especially when they lack economic security. The basic personal needs during hospitalized isolation led to the negative transformation of personal emotions.^15^ These symptoms may adversely affect the ability of isolated personnel to solve problems and lead to a decline in their life quality.^16^

The specific group categories such as “economic stress”, “isolation” and “family” are the common theme of anxiety and depression^17^, in which economic stress is particularly significant and will cause negative health problems in the future.^18^ Cauberghe et al.^19^ argue that in addition to physical health, many countries resorted to most policies about economic problems during the COVID-19 pandemic in 2019. At the same time, they believe that long-term economic depression will have an adverse impact on the mental health of isolated patients. The uncertainty caused by the COVID-19 pandemic escalates negative emotions. A cross-sectional survey involved more than 3000 patients in isolation, including COVID-19 IU, risk perception, social exclusion, perceived efficacy and negative emotions.

The results show that a positive attitude has a better protective effect on negative emotions. Taking effective measures to improve the perceived efficiency of the public and form a reasonable risk perception, increase positive attitudes and strengthen their connection with society^20^ has a positive inhibitory effect on unhealthy psychology such as anxiety and depression. DeRossett et al.^21^ believe that a higher level of negativity is positively correlated with the anxiety caused by COVID-19, while a positive response is negatively correlated with anxiety. There is some relationship between gender and anxiety & depression. Isolated women are more likely to suffer from anxiety, depression and stress than men. Alemanno et al.^22^ included 87 COVID-19 patients. After receiving Mini-Mental State Examination (MMSE), Montreal Cognitive Assessment (MoCA), Hamilton Depression Rating Scale and functional independent measurement (FIM), it shows that women need long-term psychological support and treatment to prevent the occurrence and aggravation of psychological diseases. Our study also confirms that gender (p=0.002), economic status (p=0.004) and isolation attitude (p=0.023) are the related factors of anxiety, among which economic status is the protective factor, while women and resistant attitude are the risk factors; Economic status (p=0.003) and isolation attitude (p=0.001) are the related factors of depression, among which economic status is the protective factor, while resistant attitude is the risk factor. The conclusions of this study provide clinical data for psychological intervention programs for patients with COVID-19.

### Limitations of this study:

It includes the small sample size and the lack of corresponding follow-up period. In future work, we will continue to increase the sample size and the follow-up period in order to describe the influencing factors more objectively. We hope the isolated infectious disease fever patients during the COVID-19 pandemic can receive more accurate intervention measures.

## CONCLUSION

To conclude, during the COVID-19 pandemic, hospitalized isolation patients were more likely to suffer from varying degrees of anxiety and depression than home isolation patients, and the majority of the population are women, people with bad economic conditions and people with poor attitudes toward isolation. It is necessary to provide psychological counseling and social support to the above-mentioned focus groups before isolation. If possible, we should try to urge the above-mentioned groups to isolate themselves at home to ensure their mental health and prevent the destructive impact of negative emotions on their body and mind.

### Authors’ Contributions:

**GJ and** **LL:** Designed this study, prepared this manuscript, are responsible and accountable for the accuracy and integrity of the work.

**JL and** **BW:** Collected and analyzed clinical data.

**BZ:** Data analysis, significantly revised this manuscript.
